# Imaging grain microstructure in a model ceramic energy material with optically generated coherent acoustic phonons

**DOI:** 10.1038/s41467-020-15360-3

**Published:** 2020-03-27

**Authors:** Yuzhou Wang, David H. Hurley, Zilong Hua, Thomas Pezeril, Samuel Raetz, Vitalyi E. Gusev, Vincent Tournat, Marat Khafizov

**Affiliations:** 1 Department of Mechanical and Aerospace Engineering, The Ohio State University, Columbus, OH 43210 USA; 2 Idaho National Laboratory, P.O. Box 1625, Idaho Falls, ID 83415 USA; 3 Institut Molécules et Matériaux du Mans, UMR-CNRS 6283, Le Mans Université, 72085 Le Mans, France; 4 Laboratoire d’Acoustique de l’Université du Mans, LAUM - UMR 6613 CNRS, Le Mans Université, Avenue Olivier Messiaen, 72085 Le Mans cedex 9, France

**Keywords:** Characterization and analytical techniques, Imaging and sensing

## Abstract

Characterization of microstructure, chemistry and function of energy materials remains a challenge for instrumentation science. This active area of research is making considerable strides with methodologies that employ bright X-rays, electron microscopy, and optical spectroscopy. However, further development of instruments capable of multimodal measurements, is necessary to reveal complex microstructure evolution in realistic environments. In this regard, laser-based instruments have a unique advantage as multiple methodologies are easily combined into a single instrument. A pump-probe method that uses optically generated acoustic phonons is expanding standard optical characterization by providing depth resolved information. Here we report on an extension of this method to image grain microstructure in ceria. Rich information regarding the orientation of individual crystallites is obtained by noting how the polarization of the probe beam influences the detected signal amplitude. When paired with other optical microscopies, this methodology will provide new perspectives for characterization of ceramic materials.

## Abstract (web summary)

Optically generated acoustic phonons have enabled depth resolved microstructure characterization. Here, the authors extend this method to obtain information on the orientation of individual crystallites by studying influence of probe beam polarization on detected signal amplitude.

## Introduction

Optical spectroscopies have the demonstrated capability to noninvasively characterize several aspects of advanced energy materials in realistic environments. Examples include monitoring function of organic photovoltaics using photoluminescence spectroscopy, measuring in situ changes of chemistry of fuel cell anodes using Raman spectroscopy, monitoring thermal transport across individual interfaces, and investigation of hot carrier cooling mechanisms in photovoltaics using transient absorption spectroscopy^1–4^. Additional attributes include benchtop deployment and straightforward accommodation of multiple measurement methodologies using standard optics. Adding to this list of optics-based characterization tools is a technique that uses optically generated and detected acoustic phonons to image materials in the depth direction^5,6^. This method, termed time domain Brillouin scattering (TDBS), had been first applied to characterize homogeneous semiconductors and dielectrics^7^. Applications were extended to homogeneous liquids^8^, vegetal cells^9^, and polycrystalline materials^10–12^.

Applications were further extended to depth profiling of elastic inhomogeneities in nanoporous organosilicate^13^ and ion irradiated GaAs^14^ and to three-dimensional imaging of animal cells^15^. Recent work that is closely tied to the current study involves surface scanning using TDBS to image subsurface grain boundary orientation^16^ and to resolve the position of different crystallites^17^. A review of other applications of TDBS can be found in reference^18^.

Although the velocity of longitudinal acoustic (LA) phonons are traditionally used for characterization, recent studies have demonstrated the potential of using transverse acoustic (TA) phonons to obtain additional information regarding grain microstructure^10^. Key examples include using TA and LA phonon velocities to obtain information about the first two Euler angles^17^ and using surface acoustic wave velocities to obtain the orientation of large crystallites having dimensions of a few tens of millimeters^19^. However, measurement of bulk wave velocities alone cannot resolve the third Euler angle, which rotates the crystallite around the surface normal^20–22^. In addition to measuring phonon velocities, it is expected that detailed studies of the detection process will reveal additional information about grain microstructure^10^.

Here, we systematically study the detected amplitude of TA and LA phonons as a function of probe polarization. CeO_2_ (ceria) was chosen for this study as it is a model fuel cell material owing to its mechanical stability and ionic conductivity^23,24^. Ceria is also an important surrogate material for oxide nuclear fuels^25,26^. Applying TDBS to ceria presents an excellent test case for research on advanced ceramic materials with applications in the energy industry. As discussed in detail below, our experimental results show that the detected TA phonon amplitude is a strong function of probe polarization. The probe beam polarization dependence is explained by an optoacoustic model that considers the strain associated with each acoustic mode and the probe beam polarization. Based on our finding, a method is suggested for the determination of the orientation of individual crystallites. When coupled with subsurface imaging, this approach has the prospect to fully characterize the near surface grain microstructure in three-dimensions (3D). Moreover, combining TDBS with other optical microscopies has the potential to greatly extend optics-based instrumentation science with broad application involving 3D characterization of polycrystalline ceramic materials.

## Results

### Crystallite mapping and orientation measurement

**Fig. 1:**
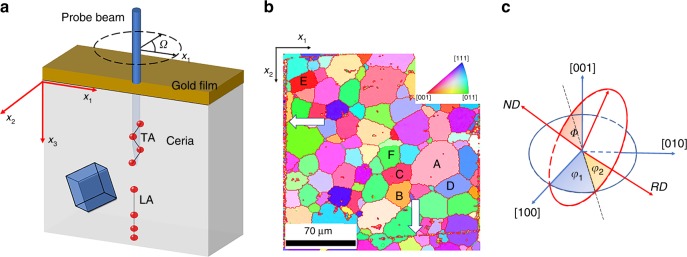
Time domain Brillouin Scattering experiments on specific crystallites in polycrystalline CeO_2_. **a** Geometry of ceria sample. **b** EBSD of sample surface. The orientation of crystallites denoted by letters A–F is given in Table 1. The platinum fiducial square, delineated by white arrows, is used to define the laboratory coordinate system. **c** The three Euler angles are used to define the crystallite orientation relative to the laboratory coordinate system. The first rotation, *φ*_1_, is about [001] and the last rotation, *φ*_2,_ is about ND.

The experimental geometry is shown in Fig. 1a. A thin gold film is coated on the sample surface to ensure strong optical absorption of the laser pump beam. A detailed description of experimental and material parameters is given in the methods section. The crystallite orientation at the surface of the sample, recorded using electron backscatter diffraction (EBSD), is shown in Fig. 1b. A platinum fiducial square, indicated by the white arrows in the EBSD image, is used to optically locate specific crystallites and to define the coordinate system. As indicated, *x*_1_ and *x*_2_ are aligned with the sides of the fiducial square and *x*_3_ is antiparallel to the surface normal. The skewed appearance of the fiducial square is related to projection issues associated with EBSD. Several representative crystallites (A–F) were identified for this measurement. The orientation of the crystallites investigated is listed in Table 1. Using metallurgical terminology, we define the direction normal to the plane (*hkl*) as the normal direction (ND), and a direction parallel to [*uvw*] as the rolling direction (RD). The relation between ND/RD and the three Euler angles is given in Fig. 1c.

**Table 1:** Summary of TDBS results on several ceria crystallites.

Label	A	B	C	D	E	F
Miller index (*hkl*)	(10,5,24)	(−1,8,26)	(−2,3,17)	(12,−8,17)	(0,0,1)	(−20,1,22)
Miller index [*uvw*]	[10,4,−5]	[26,0,1]	[27,−16,6]	[11,8,−4]	[13,1,0]	[18,−14,17]
Experimental Freq. (GHz)	93.4	94.2	96.6	88.1	99.1	88.8
	48.3	47.5	41.4	54.8	na	na
Calculated Freq. (GHz)	93.9	96.8	98.2	87.2	99.7	88.1
	48.7	44.8	40.8	55.7	38.1	60.2
Calculated *Ω*_0_	148°(FTA)	−89°(FTA)	−148.5°(FTA)	−18°(STA)	na	na
*Ω*_max_/*Ω*_min_ (model)	−29°/61°	89.5°/−0.5°	119.3°/29.3°	54°/−36°	na	na
*Ω*_max_/*Ω*_min_ (exp.)	−29.5°/60.5°	82.8°/−7.2°	122.8°/32.8°	59.4°/−30.6°	na	na
*M*/*N* ratio (model)	0.43	0.51	0.33	1.53	na	na
*M*/*N* ratio (exp.)	0.52	0.55	0.48	3.0	na	na

The calculated and measured frequencies for each crystallite compare closely.

### Time domain Brillouin scattering spectroscopy

**Fig. 2:**
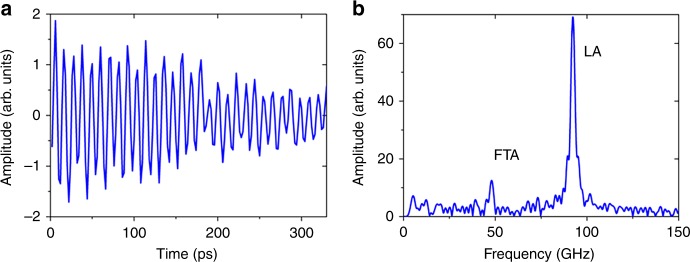
Transverse and longitudinal acoustic phonons propagating in Grain A. **a** A time domain trace of Brillouin oscillations corresponding to crystallite A. **b** Fourier amplitude spectrum of signal. Two peaks are located at 93.4 and 48.3 GHz.

An example of a reflectivity transient exhibiting strong Brillouin oscillations is shown in Fig. 2a, corresponding to crystallite A. To highlight the oscillatory features, the thermal background has been subtracted. The data exhibit two oscillatory components having different frequencies and amplitudes.

The observed Brillouin oscillations stem from optical interference between a portion of the probe that reflects from the gold film and a portion that reflects from thermoelastically generated phonons propagating in the ceria substrate (see Methods). The frequency of these oscillations is governed by *f* = *2nν*_m_*/λ*, where *n* is the refractive index, *ν*_m_ is the mode specific phonon velocity and *λ* is the photon wavelength of the probe pulse. Ceria is a cubic crystal and optically isotropic (i.e., only one electric permittivity tensor component). However, for birefringent materials, multiple frequency would be recorded for each acoustic mode^10,12^. The Fourier amplitude spectrum shown in Fig. 2b, reveals two phonon modes, located at 93.4 GHz and 48.3 GHz that are associated with the quasi longitudinal (LA) and fast quasi shear acoustic (FTA) modes, respectively. The slow quasi shear mode (STA) is not detected.

The acoustic velocities are obtained by calculating eigenvalues of the Christoffel equation using the rotated elastic stiffness tensor^16^. The Euler angles used to derive the rotated elastic stiffness tensor are *φ*_1_ = −63°, *Φ* = 25°, and *φ*_2_ = 86°. The refractive index of ceria at the probe wavelength (408 nm) was measured to be 2.57 using optical ellipsometry. The imaginary part of the index of refraction is negligible giving rise to long-lived Brillouin oscillations. Measurement of the single crystal elastic constants either requires large single crystals or methods applied to polycrystals that can measure elastic properties on length scales below the crystallite diameter. It is well known that single crystal elastic constants can be obtained by frequency-domain Brillouin scattering measurement (see, for example,^27,28^ and the references therein). The elastic constants of ceria used in this study were reported in previous work based on time domain Brillouin measurements made on several crystallites with known orientations^17^. Ceria has three independent elastic tensor components represented using engineering notation as *C*_11_, *C*_12_, and *C*_44_. We find good agreement between the experimental and calculated Brillouin frequencies for each detected mode and for each crystallite investigated as summarized in Table 1. An expression for the Brillouin frequency as a function of phonon velocity and index of refraction is given in the methods section.

### Probe polarization dependent measurements

**Fig. 3:**
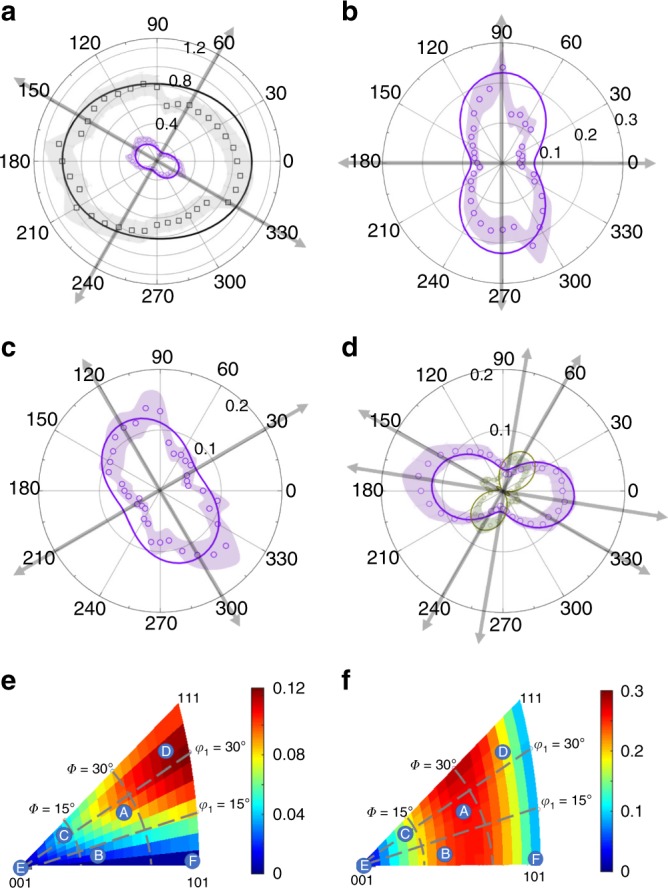
The shape and orientation of the TA amplitude profile is tied to crystallite orientation. Polar plot of LA (black), normalized FTA (violet), and normalized STA (green) amplitudes for **a** crystallite A; **b** crystallite B; **c** crystallite C; **d** crystallite D as a function of *Ω*. The crystallite orientations are summarized in Table 1. Open symbols are experimental results and solid lines are from theory (shading represents measurement error); **e** displacement ratios STA/LA; and **f** FTA/LA for different crystallite orientations (represented by a color-coded inverse pole figure).

Next, we investigate the influence of the probe beam optical polarization on the measured signal. A half-wave plate was used to rotate the probe polarization counterclockwise in steps of 10°. The power of the pump and probe beams were kept constant. The polarization angle, *Ω*, is defined as the angle between the optical polarization vector and the *x*_1_ axis (see Fig. 1a). The amplitude profiles of the detected acoustic modes for crystallite A as a function of *Ω* are shown in Fig. 3a. The major axis (connecting points of maximum amplitude) is perpendicular to the minor axis. The major/minor axes coincide with axes of mirror symmetry (represented as double-arrow lines in Fig. 3). The FTA amplitude is normalized using the largest LA amplitude. The remaining discussion will only consider the amplitude profiles of the TA modes as they exhibit a stronger dependence on probe polarization.

The normalized TA amplitude profiles for other crystallites is shown in Fig. 3b–d. The amplitude profiles indicate twofold rotation symmetry, which is consistent with previous observations of TA modes in BiFeO_3_^12^. However, it is noted that there is a slight left/right asymmetry in the experimental data. This asymmetry is not reproducible and we thus attribute it to an experimental artifact such as beam walking of the pump relative to the probe caused by rotation of the *λ*/2 plate. Using other data sets, not presented in this manuscript, we find that these asymmetries minimally influence the extracted value of *φ*_2_. For most crystallites, only the FTA mode was detected. However, in crystallite D, both the FTA and STA modes were observed. No shear modes were detected in crystallites E and F as both correspond to high symmetry directions.

## Discussion

LA acoustic pulses are generated thermoelastically in the elastically isotropic gold film (see methods). TA acoustic pulses are generated owing to mode conversion at the film/substrate interface^29–31^. The detected amplitude profile of each acoustic mode is a product of components of the strain and photoelastic tensors (see methods), the latter being a strong function of probe wavelength. A thorough literature search revealed no data for the photoelastic tensor components for ceria. Nevertheless, a set of measured profiles over various crystallites (Fig. 3) allowed us to determine the relative values of photoelastic coefficients for ceria at 408 nm. We compare the theoretical TA and LA amplitudes with the experimental results by adjusting the photoelastic ratio ([*P*_11_−*P*_12_−2*P*_44_]/*P*_12_). We obtain an optimal fit by adopting the following ratio: (*P*_11_−*P*_12_−2*P*_44_)/*P*_12_=1.2. We note that calcium fluoride, with a similar fluorite crystal structure, has (*P*_11_−*P*_12_−2*P*_44_)/*P*_12_= 1.1^32^. In the following discussion, we show that the dependence of the amplitude profiles of the TA modes on the photoelastic constants is only a function of this ratio.

Theoretical and experimental results for several crystallites are compared in Fig. 3. The theoretical amplitude profiles (see methods) are presented as solid lines. Overall, we find a good agreement between experimental and modelling results. The modelling shapes all have twofold rotation symmetry and exhibit a dipole-like feature. This is in agreement with the structural invariance after rotating the third Euler angle by 180°.

In most cases, involving crystallites aligned along low symmetry directions, only the LA and FTA modes are detected. This is confirmed using our theoretical model (see methods) that calculates relative shear strain displacement for all possible crystallite orientations (shown in Fig. 3e, f). Generally, the FTA mode has a much larger strain than the STA mode and can be observed for most orientations. Moreover, to efficiently mode convert the LA mode in the film into TA modes in the substrate require that the TA modes have a sizeable longitudinal component. For most orientations, the FTA mode has a relatively large longitudinal component as compared to the STA mode. The STA mode can only be detected in the vicinity of the (111) orientation (red region in Fig. 3e). Crystallite D is located in this region and as expected we detect both shear phonon modes. For a range of probe polarizations satisfying 40° < *Ω* < 90° and 220° < *Ω* < 270°, the amplitude of the STA mode is larger than the FTA mode. The STA also exhibits additional lobes around the minor axis.

The photoelastic tensor governs the amplitude profile of the FTA mode. Using engineering notation, the rotated photoelastic tensor is represented by a 6 × 6 matrix, $\bar P_{ij}$. The components of interest for *x*_*1*_ polarized light are $\bar P_{13},\bar P_{14},\,{\mathrm{and}}\,\bar P_{15}$ and $\bar P_{23},\,\bar P_{24},\,{\mathrm{and}}\,\bar P_{25}$ for *x*_2_ polarized light. Examination of these terms reveals that they have the following form:

1 $${\bar{P}}_{13} = 	\, P_{12} + \Delta _1 \times (P_{11} - P_{12} - 2P_{44}) \\ {\bar{P}}_{14} = 	\, \Delta _2 \times (P_{11} - P_{12} - 2P_{44}) \\ {\bar{P}}_{15} =	 \, \Delta _3 \times (P_{11} - P_{12} - 2P_{44})$$

where Δ_*i*_(*φ*_1_, *Φ*, *φ*_2_) are constants defined by the crystallite orientation. $\bar P_{23},\,\bar P_{24},\,{\mathrm{and}}\,\bar P_{25}$ share the same form, although Δ_*i*_ terms are different. The fact that all terms in Eq. (1) have a common factor *P*_11_−*P*_12_−2*P*_44_ shows its significance in the detection process. Its value physically quantifies the coupling strength between the shear strains of the acoustic phonon modes and the electric field of the polarized probe beam. Therefore, the detectability of TA phonon is strongly related to the amplitude of this factor. In reality, by rotating the probe polarization, the second term on the right-hand side of $\bar P_{13}$ has at most a comparable magnitude to that of the first term. Therefore, rather than giving the values of *P*_*ij*_, we fit the ratio (*P*_11_−*P*_12_−2*P*_44_*)/P*_12_. From this observation, it is possible to study *P*_12_ alone if the crystallite has an orientation where the Δ_*i*_ terms are very small. A similar experiment has been undertaken to study the evolution of *P*_12_ in proton irradiated GaAs^33^. Here, we define the anisotropy of the photoelastic tensor ${\upalpha}_{\mathrm{r}} = 2P_{44}/(P_{11} - P_{12})$, in analogy to the Zener ratio for the elastic tensor. In the case of photoelastic isotropy, *α*_r_ = 1, only $\bar P_{13} = P_{12}$ is nonzero and the detected amplitude does not depend on probe polarization. Photoelastic isotropy has been observed in several alkali halides at certain wavelength where optical birefringence does not depend on the strain^34^. Thus, the dependence on the optical polarization of the probe beam, shown in Fig. 3, is a consequence of photoelastic anisotropy. It is expected that as the photoelastic tensor becomes more anisotropic, *or* as *α*_r_ becomes more distinct from unity, the optical polarization dependence of the detected LA and TA phonon amplitudes becomes more pronounced.

The shape of *m*th mode amplitude profile can be written as a function of the strain induced change in the dielectric permittivity. Using engineering notation, the relative reflectivity change can be expressed as^35^

2 $$\frac{{\delta R}}{R} \propto \Delta \varepsilon _1{\mathrm{cos}}^2{\it{\Omega}} + \Delta \varepsilon _2{\mathrm{sin}}^2{\it{\Omega }} + \Delta \varepsilon _6{\mathrm{sin}}2{\it{\Omega }}$$

Modulation of the dielectric permittivity, Δ*ε*_1_, Δ*ε*_2_, and Δ*ε*_6_, are calculated for a given crystallite orientation. Equation (2) can be transformed into a following simplified form for the *m*th acoustic mode

3 $$\left( {\frac{{\delta R}}{R}} \right)_m \propto N_m + M_m \times {\mathrm{sin}}(2{\it{\Omega }} + {\it{\Omega }}_{0,m})$$

where

4 $$\tan \left( {{\it{\Omega }}_{0,m}} \right) = \frac{{\Delta \varepsilon _{1,m} - \Delta \varepsilon _{2,m}}}{{2\Delta \varepsilon _{6,m}}}$$

and

5 $$\begin{array}{*{20}{l}} {N\left( {\varphi _1,{\it{\Phi }},\varphi _2} \right)} \hfill & = \hfill & {\frac{{\Delta \varepsilon _1 + \Delta \varepsilon _2}}{2}} \hfill \\ {M\left( {\varphi _1,{\it{\Phi }},\varphi _2} \right)} \hfill & = \hfill & {\sqrt {\left( {\frac{{\Delta \varepsilon _1 - \Delta \varepsilon _2}}{2}} \right)^2 + (\Delta \varepsilon _6)^2} } \hfill \end{array}$$

From Eq. (3), it is straightforward to see that the polarization dependence has a dipole shape and that it has the largest amplitude (*N*+*M*) at ${\it{\Omega}} _{\mathrm{max}} = \frac{{\pi - 2{\it{\Omega }}_0}}{4}$ and smallest amplitude (|*N*−*M*|) at ${\it{\Omega}} _{\mathrm{min}} = \frac{{ - \pi - 2{\it{\Omega }}_0}}{4}$ when *N* > *M* (crystallites A, B, C). The theoretical amplitude profiles using Eq. (3) (solid line) are compared with experimental data in Fig. 3a–d. Although the shape of the theoretical amplitude profiles closely follows the experimental data it is noted that the magnitude of the amplitude profile in some cases is over/under estimated. This is due to the fact that we used a global fit, using all of the data acquired, to extract the photoelastic ratio. In the case where *N* < *M* (STA mode in crystallite D), the relative reflectivity given in Eq. (3) will experience a crossover from positive to negative values during rotation, and the absolute value will reach a minimum value at angles defined by $N + M \times {\mathrm{sin}}\left( {2{\it{\Omega }} + {\it{\Omega }}_0} \right) = 0$. As a result, an additional set of lobes are observed near the minor axis of crystallite D shown in Fig. 3d. In both cases (*N* > *M* and *N* < *M*) the responses are mirror symmetric along the major axis defined by *Ω*_0_ and have twofold rotation symmetry.

It is important to note that the parameter *Ω*_0_ is a strong function of crystallite orientation (*φ*_1_, *Φ*, *φ*_2_). Consider the FTA mode in crystallite A as an example. From Eq. (4) we calculate *Ω*_0,2_ = 148°, indicating min/max amplitudes of the FTA mode to be along 61°/−29° directions. This agrees very well with the result presented in Fig. 3a. The same calculation was also conducted on crystallites B, C, D and is summarized in Table 1 (negative angles are located in the third and fourth quadrants). Experimental results and model prediction show close agreement (all cases are within 7°).

**Fig. 4:**
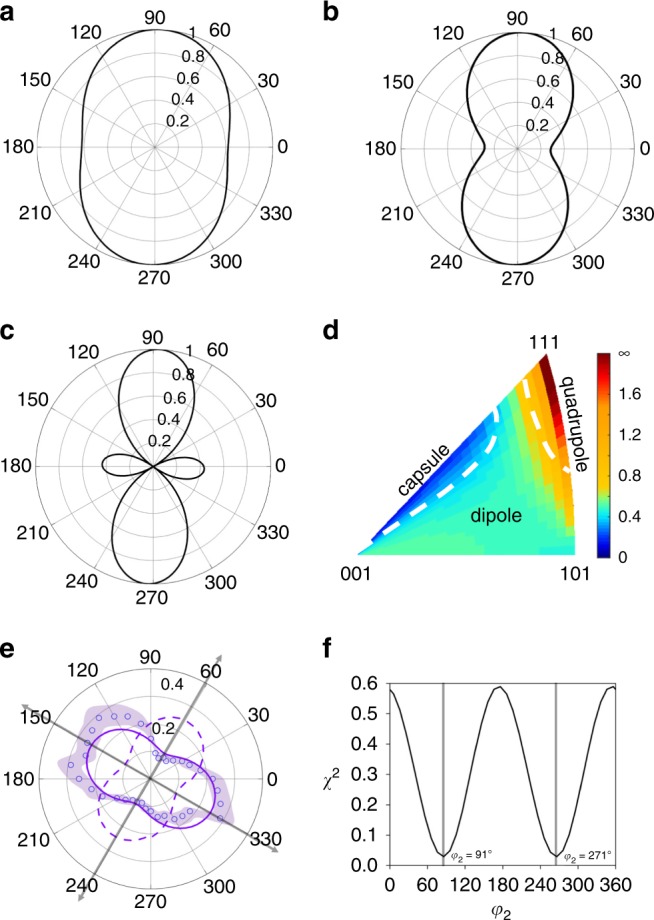
Impact of grain orientation on the FTA amplitude profile. **a** capsule shape (*N* » *M*); **b** dipole shape (*N* > *M*); **c** quadrupole shape (*N* < *M*); **d** ratios of *M* and *N* and corresponding shapes projected onto a color-coded inverse pole figure; **e** comparing two profiles that have the same values of *φ*_1_ and *Φ* but different values for *φ*_2_ (solid and dashed line). The solid line corresponds to the best fit to the experimental data (circles); **f** the sum of the square of the differences between experiment and theory versus *φ*_2_.

Next, we discuss how the Euler angles affect the amplitude profiles of the FTA mode. The shape is determined by the first two Euler angles, *φ*_1_ and *Φ*. We categorize the shapes into three groups: capsule shape, dipole shape, and quadrupole shape. These shapes are presented in Fig. 4a–c, respectively. In the first case, the shape does not have an eminent dip near the minimum, which corresponds to *N* ≫ *M*. The second group corresponding to *N* > *M*, is the most common. The last group is the least common and corresponds to *N* < *M*. In Fig. 4d, we plot the *M* and *N* ratios and their corresponding shape onto a color-coded inverse pole figure for FTA mode. The majority of the orientations produce a dipole shape, in agreement with our experimental results. It is also noted that the third Euler angle *φ*_2_ by definition rotates the crystal around *x*_3_ axis (ND) and thus does not change the dipole shape, only its orientation. This point is illustrated in Fig. 4e, where two profiles that have the same values of *φ*_1_ and *Φ* but different values for *φ*_2_ (solid and dashed line) are compared.

The result of these findings suggests a method for the complete crystallite orientation using TDBS. The modulated optical permittivity components, $\Delta \varepsilon _{k,i}$, implicitly depend on three angles (*φ*_1_, *Φ*, *φ*_2_) and elasticity (*C*_*ij*_) as well as photoelasticity $\left( {P_{12}\,{\mathrm{and}}\,P_{11} - P_{12} - 2P_{44}} \right)$. In principle, all three angles can be obtained from measurement of Brillouin frequencies and the FTA amplitude profile. Applying this approach to grain C, we obtain *φ*_1_ = −67°, and *Φ* = −23° from the measured Brillouin frequencies. In the next step we calculate the sum of the square of the differences, *χ*^2^, between the experimental and theoretical amplitude profiles versus *φ*_2_ (Fig. 4f). The minimum value of *χ*^2^ corresponds to *φ*_2_ =  91. These angles agree closely with the values obtained from EBSD, *φ*_1_ = −64°, and *Φ* = −25° and *φ*_2_ = 85°. The shape of *χ*^2^ is fairly steep and exhibits a narrow minimum, suggesting high sensitivity to *φ*_*2*_. It is noted the procedure sketched above requires knowledge of the photoelastic ratio, *P*_12_/(*P*_11_−2*P*_44._). Because information on the photoelastic constants for most materials is scarce, the photoelastic ratio can be obtained by making measurements on several crystallites with known orientation and fitting predicted polarization plots to the measured ones by varying the photoelastic ratio. In addition, although the STA mode is not required to uniquely determine all three Euler angles, it is expected that the accuracy of this approach would be even higher for orientations for which the STA mode is generated and detected.

To place this new application of TDBS in the proper perspective it is important to understand current limitations. First, this technique is only appropriate for materials that are transparent or partially transparent at the probe wavelength. In addition, for materials that are transparent at the pump wavelength, a thin metallic film must be applied to ensure strong optical absorption. The second limitation is related to spatial resolution. Currently the lateral spatial resolution of the technique is limited by the overlap of the foci of the pump and the probe laser pulses. Using far-field focusing techniques, a spot size of 1 µm is easily achievable, and spot sizes of ~100 nm can be obtained by employing near-field focusing techniques^36,37^. Although these values are at least an order of magnitude larger than that of scanning electron microscope-based methods, this approach does not require serial sectioning to obtain depth resolved information and provides volumetric information over a larger depth than accessible using focused ion beam liftout techniques. Lastly, this technique uses anisotropy in material's property to extract crystallite orientation. As a consequence, the sensitivity to orientation increases with increasing elastic and photoelastic anisotropy.

In conclusion, we applied TDBS to characterize grain microstructure in a polycrystalline ceria sample. It was shown that for crystallites oriented along low symmetry directions, both LA and TA modes are generated and detected. The generation of the TA modes is due to mode conversion of the LA mode at the interface between the elastically isotropic gold transducer film and the elastically anisotropic substrate. The TA amplitude profiles exhibit a strong dependence on the direction of the probe polarization vector. Using an optoacoustic model that accounts for elastic and photoelastic anisotropy, it is shown that the shape of the amplitude profile for the FTA mode falls into one of three categories (capsule, dipole, quadrupole). A method was suggested to extract all three Euler angles by using the orientation of the major axis of the FTA amplitude profile and the velocities of the detected phonon modes. While outside the scope of the current manuscript, future work will involve refinement of this approach by considering the shape, orientation and relative magnitude of the amplitude profiles for all of the detected phonon modes.

The results presented in this manuscript are important for a broad class of applications involving 3D imaging of polycrystalline ceramic materials relevant to the energy industry. Energy materials are routinely exposed to extremes in temperature, pressure and radiation. As a consequence, further materials development will require instruments that can record multiple aspects of material performance in realistic environments. The current work involving the development of non-invasive imaging capability of 3D microstructure is an important step in this direction. Future work aimed at combining 3D imaging using TDBS with other optical microscopies will provide synthesis scientists with essential information regarding in-situ and/or in-operando changes in material structure and function.

## Methods

### TDBS measurement

The sample investigated was a 3 × 4 × 5 mm sintered ceria pellet, purchased from AlfaAesar. The as-received sample was annealed at 1600 °C for 4 h in an oxygen rich environment to prevent reduction and promote grain growth. The average grain size of the annealed sample was 50 µm. The sample was polished to be optically flat and a fiducial platinum square measuring 500 × 500 µm^2^ was deposited using a focused ion beam. The last preparation step involved sputter coating a thin (~7 nm) gold transducer film to ensure strong optical absorption and to promote acoustic mode conversion at the film/substrate interface. The orientation of the crystallites at the sample surface were imaged using EBSD.

An optical pump-probe setup was used to measure the propagation of acoustic phonons in the ceria substrate. A mode-locked Ti-sapphire laser pump beam of pulse duration ~1 ps, repetition rate 76 MHz and wavelength 816 nm is focused onto the sample with a spot size of ~2 µm and a fluence ~0.01 mJ cm^−2^. The pump beam is amplitude modulated at 1 MHz using an acousto-optic modulator. A time-delayed, normal incident 408 nm probe with similar fluence and beam waist as the pump beam is focused onto the sample in the same location as the pump beam. The time delay was imposed by a mechanical shaker with a maximum scan time of ~350 ps. Small changes in the signal intensity governed by the photoelastic effect are demodulated using a lock-in amplifier. To ensure strong photoelastic coupling, the output wavelength of the laser is selected such that the probe photon energy is close to the ceria band gap^38^. Optical attenuators are used to control the incident powers of the pump and probe beams. A half-wave plate is placed in the probe leg to rotate the probe polarization. The sample is placed on a 2D translation stage, with micron resolution, to precisely locate the grains.

The material parameters for the elastically isotropic nanocrystalline gold film are taken as: ${\rho} = 19.32\,{\mathrm{g}}\,{\mathrm{cm}}^{ - 3},\,{\mathrm{and}}\,C_{11} = 220\,{\mathrm{GPa,}}\,C_{44} = 26\,{\mathrm{GPa,and}}\,\beta ^{\prime} = 42 \times 10^{ - 6}K^{ - 2}$ ^32^, where ${\rho},C_{ij}{\mathrm{,and}}\,\beta^{\prime}$ are the density, the elastic constants and the coefficient of thermal expansion, respectively. Elastic constants and density of single crystal ceria are taken as: $C_{11} = 451\,{\mathrm{GPa,}}\,C_{12} = 119\,{\mathrm{GPa}},C_{44} = 66\,{\mathrm{GPa,}}\,\rho = {\mathrm{7}}{\mathrm{.22}}\,{\mathrm{g}}\,{\mathrm{cm}}^{ - 3}$^17^.

### Optoacoustic model

The acoustic pulses propagating in ceria are thermoelastically generated in the gold film. Absorption of femtosecond laser pulses in gold is known to create a non-equilibrium distribution of overheated charge carriers, which transfer energy to the lattice (to the thermal phonons) in a characteristic time *τ*_*E*_ ≈ 0.5 ps^39^. Because the thickness of the gold film (*H* =7 nm) is much shorter than the diffusion length of the non-equilibrium electrons, the gold film is heated uniformly in the depth direction^39^. The strain pulses in ceria have a characteristic duration equal to $\tau _a = \frac{{2H}}{{v_{Au}}} \approx 4\,{\mathrm{ps}}$, and are composed of two segments of the same shape but opposite polarity. The acoustic waves propagating in the direction of ceria substrate contribute to the leading edge of the strain pulses, whereas waves that are reflected from the mechanically free surface of the gold film contribute to the trailing edge. The characteristic duration of the emitted strain pulse *τ*_*a*_ is much longer than both the pump laser pulse duration and *τ*_*E*_. Thus, the heating and the accompanying stress initiation can be considered as instantaneous. Cooling of the gold takes place on a time scale exceeding 50 ps, thus thermo-elastic stresses associated with cooling provide negligible contribution to the high frequency acoustic waves monitored in our experiments (see Fig. 2b). The strain pulses emitted in ceria (*x*_3_ ≥ 0) can thus be modeled accurately using the following spatial profiles:

6 $$\eta _{i3}\left( {x_3,t} \right) = - \frac{{A_{im}}}{2}\left\{ {\begin{array}{*{20}{c}} {\left[ {\theta \left( {x_3 - v_mt} \right) - \theta \left( {x_3 - v_mt + H} \right)} \right] - } \\ {\left[ {\theta \left( {x_3 - v_mt + H} \right) - \theta \left( {x_3 - v_mt + 2H} \right)} \right]} \end{array}} \right\}$$

In Eq. (6), *θ* represents the sign function, *v*_*m*_ is the velocity of the acoustic mode and *m* is a summation index accounting for the three acoustic modes. The shear polarized modes are launched owing to mode conversion at the film/ceria interface (*x*_3_ = 0) of the purely LA pulse generated in the gold film. The index *i* in Eq. (6) indicate one of the three nonzero strain components in the emitted plane acoustic waves. The amplitudes *A*_*im*_ of the strain components are all proportional to the photo-induced temperature rise in the gold film, its thermal expansion coefficient and mode conversion coefficients. The mode conversion coefficients are a function of the rotated elastic stiffness tensor for each crystallite and are obtained from the elastic boundary conditions at the film/substrate interface^29,35^. Owing to good acoustic matching between gold and ceria, we have neglected the emission of the acoustic waves in ceria at times *t* > *τ*_*a*_ caused by multiple reflections within the gold film. We estimated that the displacement amplitude of acoustic waves emitted at *t* > *τ*_*a*_ is < 15% of the incident wave amplitude for all possible orientations of the ceria crystallites.

Governed by the photoelastic effect, the strain field in ceria (described by Eq. (6)) induces a local change in permittivity, $\Delta \epsilon_{ij}={\bar{p}}_{ijkl}\eta_{kl}$. Similar to the elastic stiffness tensor, the rotated photoelastic tensor, $\bar p_{ijkl}$, is obtained using the Euler angles for each crystallite. Owing to the small amplitude of the acoustic pulses, the optical reflection coefficients associated with this change in permittivity can be evaluated using the single-scattering approximation^40^. The optical reflection coefficients *r*_*ij*_ for the *j*th component of the incident electric field into the *i*th component of the reflected electric field is given by:

7 $$r_{ij} = \int_0^\infty {\Delta {\it{\epsilon }}_{ij}} \left( {x_3,t} \right)e^{ - 2ik_pnx_3}dx_3$$

where *k*_*p*_ and *n* denote the wavenumber of the probe light in vacuum and the refractive index, respectively. For the 408 nm probe light, the imaginary portion of the refractive index is set to zero. At times *t* > *τ*_*a*_, when the acoustic field in Eq. (6) is completely launched/located inside ceria, integration of Eq. (7) can be done explicitly, demonstrating that light scattering, as expected, is induced not by a homogeneous strain but by strain gradients:

8 $$r_{ij} = \frac{{2\bar p_{ijk3}}}{{ik_{\mathrm{p}}n}}e^{2ik_pnH}\left[ {\sin \left( {k_{\mathrm{p}}nH} \right)} \right]^2A_{km}e^{ - 2ik_{\mathrm{p}}nv_mt}$$

Here, the phase shift of the probe light scattered by each acoustic mode grows proportionally with penetration depth *v*_*m*_*t* in ceria $( {\varphi _m = 2{\mathrm{Re}}( {k_{\mathrm{p}}} )nv_mt} )$. Equation (8) also illustrates that only phonons with wave-vectors equal to twice the wave-vector of the optical probe light in ceria are detected. Two components of the probe beam, one reflected from the film surface and one reflected from the phonon propagating in the ceria sample, mix at the photodetector. This mixing sets up Brillouin oscillations with a characteristic frequency $\omega _{B,m} = \frac{{\partial \varphi _m}}{{\partial t}} = 2{\mathrm{Re}}( {k_p} )nv_m$.

## Data Availability

All data generated in this study are available from the corresponding authors upon request.

## References

[1] Miller S (2008). Investigation of nanoscale morphological changes in organic photovoltaics during solvent vapor annealing. J. Mater. Chem..

[2] Cheng Z, Liu M (2007). Characterization of sulfur poisoning of Ni–YSZ anodes for solid oxide fuel cells using in situ Raman microspectroscopy. Solid State Ion-..

[3] Hurley D, Khafizov M, Shinde S (2011). Measurement of the Kapitza resistance across a bicrystal interface. J. Appl. Phys..

[4] Fu J (2017). Hot carrier cooling mechanisms in halide perovskites. Nat. Commun..

[5] Thomsen C, Grahn HT, Maris HJ, Tauc J (1986). Surface generation and detection of phonons by picosecond light pulses. Phys. Rev. B.

[6] Thomsen C, Grahn HT, Maris HJ, Tauc J (1986). Picosecond interferometric technique for study of phonons in the brillouin frequency range. Opt. Commun..

[7] Grahn HT, Maris HJ, Tauc J (1989). Picosecond ultrasonics. IEEE J. Quantum Electron.

[8] Shelton LJ, Yang F, Ford WK, Maris HJ (2005). Picosecond ultrasonic measurement of the velocity of phonons in water. Phys. Stat. Sol. (b).

[9] Rossignol C (2008). In Vitro picosecond ultrasonics in a single cell. Appl. Phys. Lett..

[10] Lejman M (2014). Giant ultrafast photo-induced shear strain in ferroelectric BiFeO_3_. Nat. Commun..

[11] Nikitin SM (2015). Revealing sub-μm and μm-scale textures in H2O ice at megabar pressures by time-domain Brillouin scattering. Sci. Rep..

[12] Lejman, M. et al. Ultrafast acousto-optic mode conversion in optically birefringent ferroelectrics. *Nat. Commun*. **7**, 12345 (2016). 10.1038/ncomms12345 PMC4980447 27492493

[13] Mechri C (2009). Depth-profiling of elastic inhomogeneities in transparent nanoporous low-k materials by picosecond ultrasonic interferometry. Appl. Phys. Lett..

[14] Steigerwald A (2009). Semiconductor point defect concentration profiles measured using coherent acoustic phonon waves. Appl. Phys. Lett..

[15] Danworaphong S (2015). Three-dimensional imaging of biological cells with picosecond ultrasonics. Appl. Phys. Lett..

[16] Khafizov M (2016). Subsurface imaging of grain microstructure using picosecond ultrasonics. Acta Mater..

[17] Wang Y (2019). Nondestructive characterization of polycrystalline 3D microstructure with time-domain Brillouin scattering. Scr. Mater..

[18] Gusev VE, Ruello P (2018). Advances in applications of time-domain Brillouin scattering for nanoscale imaging. Appl. Phys. Rev..

[19] Smith, R. et al. Spatially resolved acoustic spectroscopy for rapid imaging of material microstructure and grain orientation. *Meas. Sci. Technol.* **25**, 5 (2014).

[20] Sinogeikin SV, Bass JD (2000). Single-crystal elasticity of pyrope and MgO to 20 GPa by Brillouin scattering in the diamond cell. Phys. Earth Planet. Inter.

[21] Weidner DJ, Carleton HR (1977). Elasticity of coesite. J. Geophys. Res..

[22] Graff KF (1991). Wave Motion in Elastic Solids.

[23] Godickemeier M, Gauckler L (1998). Engineering of solid oxide fuel cells with ceria-based electrolytes. J. Electrochem. Soc..

[24] Marrocchelli D, Bishop SR, Tuller HL, Yildiz B (2012). Understanding chemical expansion in non-stoichiometric oxides: ceria and zirconia case studies. Adv. Funct. Mater..

[25] Weber WJ (1984). Alpha-irradiation damage in CeO_2_, UO_2_ and PuO_2_. Radiat. Eff..

[26] Khafizov M (2014). Thermal conductivity in nanocrystalline ceria thin films. J. Am. Ceram. Soc..

[27] Ryu M, Cang Y, Wang Z, Fytas G, Morikawa J (2019). Temperature-dependent thermoelastic anisotropy of the phenyl pyrimidine liquid crystal. J. Phys. Chem. C..

[28] Tan J-C (2012). Exceptionally low shear modulus in a prototypical imidazole-based metal-organic framework. Phys. Rev. Lett..

[29] Pezeril T (2007). Generation and detection of plane coherent shear picosecond acoustic pulses by lasers: experiment and theory. Phys. Rev. B.

[30] Bienville, T. & Perrin, B. in World congress on ultrasonics (Paris; 2003).

[31] Pezeril T (2016). Laser generation and detection of ultrafast shear acoustic waves in solids and liquids. Opt. Laser Technol..

[32] Handbook of Chemistry and Physics. 99th edn. (CRC Press, 2018).

[33] Baydin, A., Krzyzanowska, H., Gatamov, R., Garnett, J. & Tolk, N. The photoelastic coefficient P12 of H+ implanted GaAs as a function of defect density. *Sci. Rep*. **7**, 15150 (2017). 10.1038/s41598-017-14903-x PMC5680326 29123121

[34] Rahman, A. & Iyengar, K.S. Dispersion of piezo-optic constants of some alkali halides in the ultraviolet region-I: determination of absolute values. *Acta Crystallogr.* **A26**, 128–133 (1970).

[35] Scherbakov AV (2013). Picosecond opto-acoustic interferometry and polarimetry in high-index GaAs. Opt. Express.

[36] Vertikov A, Kuball M, Nurmikko AV, Maris HJ (1996). Time‐resolved pump‐probe experiments with subwavelength lateral resolution. Appl. Phys. Lett..

[37] Siry P, Belliard L, Perrin B (2003). Picosecond acoustics with very high lateral resolution. Acta Acust. U. Ac..

[38] Miller JK (2006). Near-bandgap wavelength dependence of long-lived traveling coherent longitudinal acoustic phonons in GaSb-GaAs heterostructures. Phys. Rev. B.

[39] Gusev VE, Wright OB (1998). Ultrafast nonequilibrium dynamics of electrons in metals. Phys. Rev. B.

[40] Gusev V (1996). Laser hypersonics in fundamental and applied research. Acta Acust. U. Ac..

